# Progressive Resistance Exercise Training and Physical and Behavioural Health Components in Adults with Intellectual Disabilities: A Repeated Time-Series Design

**DOI:** 10.3390/sports14080359

**Published:** 2026-08-19

**Authors:** Kirsten I. de Oude, Roy G. Elbers, Alyt Oppewal, Dederieke A. M. Maes-Festen

**Affiliations:** 1 Department of General Practice, Intellectual Disability Medicine Research, Erasmus MC, University Medical Center Rotterdam, 3000 CA Rotterdam, The Netherlands; g.elbers@erasmusmc.nl (R.G.E.); a.oppewal@erasmusmc.nl (A.O.); d.maes-festen@erasmusmc.nl (D.A.M.M.-F.); 2 Academic Collaborative Research Center Healthy Ageing and Intellectual Disabilities, 3000 CA Rotterdam, The Netherlands

**Keywords:** strength training, challenging behaviour, physical fitness, developmental disabilities, body composition, exercise intervention

## Abstract

This study, part of the PRET-study (NCT06579898), investigated changes in body composition, physical fitness, sarcopenia, activities of daily living (ADL), challenging behaviour, and falls in adults with intellectual disabilities (ID) following participation in a 24-week progressive resistance exercise training (PRET) intervention. A repeated-time-series design with one study group was used. We used multilevel linear regression models and non-parametric tests to investigate changes in body composition (bioelectrical impedance), physical fitness (ID-fitscan), sarcopenia, ADL (Barthel Index, Lawton IADL scale), challenging behaviour (Aberrant Behaviour Checklist), and falls. Thirty-six adults with mild to moderate ID (mean age: 55.6 ± 12.9 years; 52.8% male) participated and 26 completed the intervention. Intention-to-treat and per-protocol analyses showed statistically significant improvements in physical fitness and challenging behaviour. As these analyses concerned multiple secondary outcomes from a study powered for cardiovascular risk factors, these findings should be considered exploratory. Intention-to-treat analyses showed improvements in the five-times chair stand (−2.40 s, 95% CI [−4.06; −0.75]), and reductions in irritability (−1.10, 95% CI [−2.21; −0.00]) and inappropriate speech (−0.75, 95% CI [−1.22; −0.28]). No statistically significant changes were observed for body composition, ADL, or falls and no cases of sarcopenia were identified. The findings suggest that participation in PRET may be associated with improvements in lower body functional muscular endurance and challenging behaviour.

## 1. Introduction

Physical activity levels are generally low in adults with intellectual disabilities (ID) [1,2], with only 9% reaching adequate levels for health benefits [3]. This inactivity contributes to low physical fitness, which is associated with several health conditions in this population, such as cardiovascular disease [4]. Cardiovascular risk factors (CVRFs) are also highly prevalent in older adults with ID. For example, de Winter et al. [5,6] reported hypertension in 53% of older adults with ID, obesity in 25.6%, hypercholesterolemia in 23%, diabetes in 13.7%, and metabolic syndrome in 44.7% of older adults with ID.

This study is part of the PRET study (Progressive Resistance Exercise Training) (ClinicalTrials.gov, NCT06579898) [7], which investigated the effect of PRET on CVRFs in adults with ID. The primary outcome of the PRET study was the change in CVRFs. This report presents the changes in a range of secondary outcomes, including body composition, physical fitness, sarcopenia, activities of daily living (ADL), challenging behaviour, and falls after participation in PRET.

Low levels of physical activity can result in sarcopenia, defined as the progressive and generalised loss of skeletal muscle mass and strength [8]. In older adults with ID, sarcopenia is more prevalent and may occur at a younger age compared to the general population [9,10]. Sarcopenia can lead to reduced mobility and limitations in activities of daily living (ADL), increasing dependency on caregivers. Furthermore, falls are common in older adults with ID and are associated with serious injuries and loss of independence [10,11,12]. The combination of low physical fitness and sarcopenia increases fall risk [12]. These previous findings highlight the importance of addressing physical fitness and sarcopenia in adults with ID.

In addition to physical health outcomes, behavioural aspects are also closely linked to physical activity levels in this population [9,10]. Challenging behaviour is a significant concern in adults with ID, often leading to reduced quality of life, increased caregiver burden, and limited participation in daily activities [13]. While behavioural or psychopharmacological interventions are commonly used, there is growing interest in exercise as a supportive strategy. A systematic review by Ogg-Groenendaal et al. [10] found that participation in physical activity interventions was associated with reductions in challenging behaviour in individuals with ID. Furthermore, Smith and Merwin (2021) highlighted that physical activity and exercise can improve emotional regulation, executive functioning, and stress resilience in individuals with mental health disorders [14]. Although only a limited number of studies have examined exercise interventions and behaviour in this population, the findings highlight the potential of exercise interventions in managing challenging behaviour in adults with ID.

Despite the known benefits of physical activity and exercise, adults with ID often require additional support and access to facilities to engage in and maintain an active lifestyle [15,16,17]. Therefore, intervention strategies must be tailored to the specific needs and abilities of this population. Weterings et al. [18] developed a resistance training exercise set for adults with ID (RESID) and demonstrated that using RESID in a PRET intervention was feasible in adults with mild or moderate ID and CVRFs [19]. Furthermore, previous studies in individuals with ID investigating the effects of resistance training have shown its benefits on body composition and physical fitness, including improvements in strength, balance, and body mass index [20,21]. However, evidence regarding the effects of resistance training on clinically relevant outcomes such as sarcopenia, activities of daily living, challenging behaviour, and falls remain scarce. These outcomes are highly relevant because they are directly related to functional independence, quality of life, participation, and long-term health in adults with ID. While the PRET study primarily focused on cardiovascular risk factors, it was also designed to examine broader health and functional outcomes. The present paper reports on these outcomes, which have received limited attention in the literature. Therefore, the aim of this study was to investigate changes in body composition, physical fitness, sarcopenia, ADL, challenging behaviour, and falls following participation in PRET in adults with ID.

## 2. Methods

### 2.1. Study Design

We used a repeated time series design with one study group [7]. The study period was divided into a 12-week baseline period, a 24-week intervention period (PRET programme), and a 12-week follow-up period. We used three baseline measurements for control comparisons. A minimum of three measurements is needed to establish a valid baseline and to filter out the natural variability that may exist in these parameters without applying any intervention [22]. A sample size calculation was performed for the primary outcomes of the PRET study and is described in the published study protocol [7]. The present manuscript reports secondary outcome analyses of this study.

The study was conducted within the Academic Collaborative Research Center Healthy Ageing and Intellectual Disabilities (HA-ID). The HA-ID Research Center is a collaboration between the Intellectual Disability Medicine Research group of the Erasmus MC, University Medical Center Rotterdam and three care providers in the Netherlands specialised in the care for adults with ID: Abrona (Huis ter Heide), Amarant (Tilburg), and Ipse de Bruggen (Zoetermeer). The PRET study is registered in ClinicalTrials.gov (NCT06579898). Written informed consent was obtained from all participants or their legal representatives.

### 2.2. Participants

**Figure 1:**
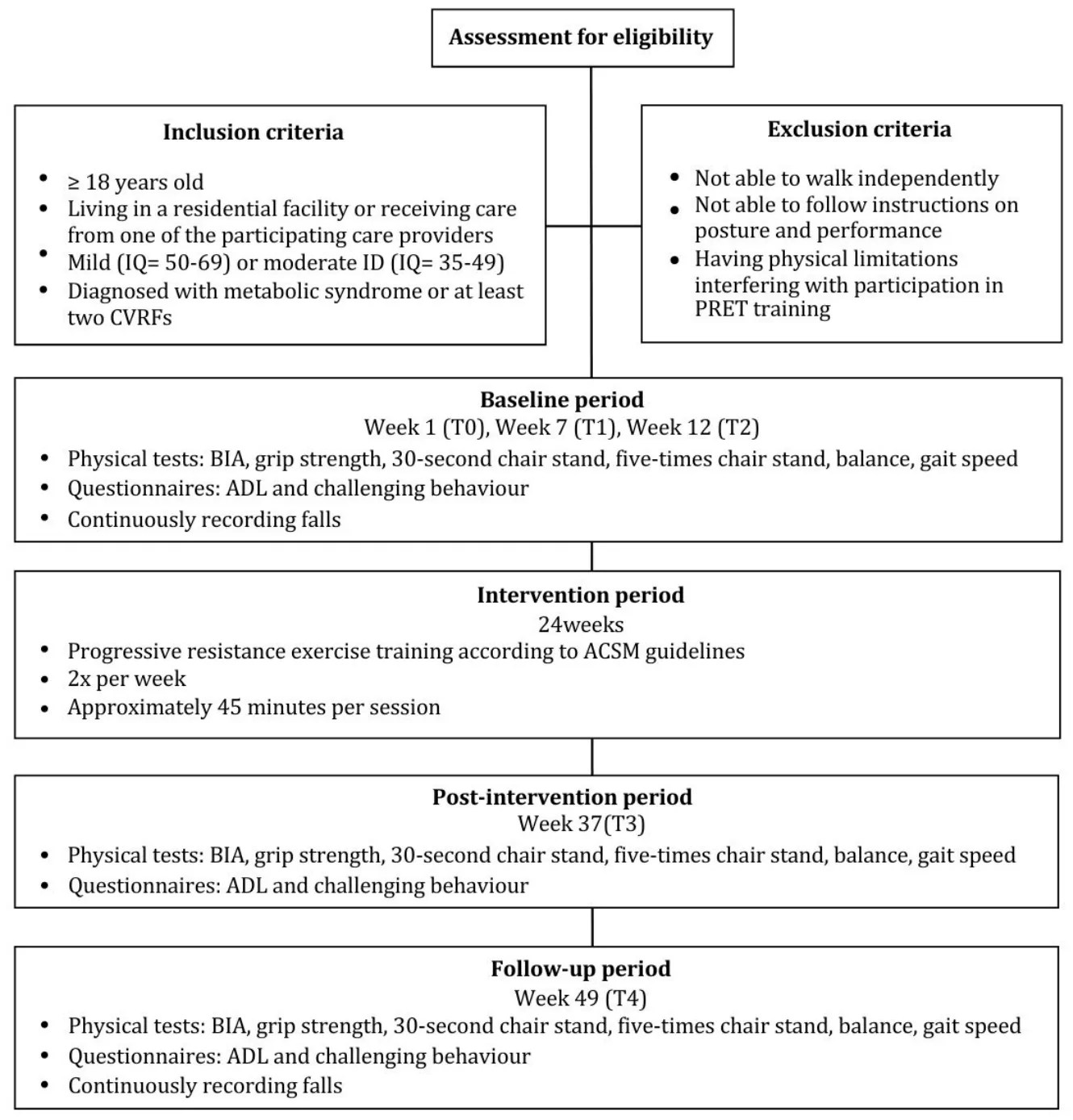
Study overview. ACSM: American College of Sports Medicine; CVRF: cardiovascular risk factors; ID: intellectual disability; IQ: intelligence quotient; BIA: bioelectrical impedance analysis; ADL: activities of daily living.

Figure 1 presents an overview of the study procedures. The PRET study included participants who were living in a residential facility or received care from one of the participating care providers. Participants had to be 18 years or older with a mild (IQ = 50–69) or moderate (IQ = 35–49) ID and had to be diagnosed with metabolic syndrome or at least two of the following CVRFs: hypertension, hypercholesterolemia, diabetes mellitus type 2 (DM2), or obesity. More details about the selection of participants and consent procedure have been published in the study protocol [7].

### 2.3. Measurements

Medical staff collected the participant characteristics of age, sex, and aetiology and level of ID from medical records. Outcome measures were assessed three times during the baseline period (T0—week 1, T1—week 7, T2—week 12), following the intervention period (T3—week 37) and at 12 weeks follow-up (T4—week 49). Measurements consisted of bioelectrical impedance analysis (BIA), physical fitness tests, questionnaires and a fall agenda. All physical measurements were conducted in a standardised order (physical fitness, BIA). Participants were not assessed in a fasted state. To reduce variability, measurements were scheduled at the same location, time, and day of the week. Full details on the outcome measures are reported elsewhere [7].

#### 2.3.1. Body Composition

We used BIA (Tanita Body Composition Analyzer MC-780, Tanita, Tokyo, Japan) to measure weight (kg), Body Mass Index (BMI), fat mass (kg and %), and skeletal muscle mass (SMM; kg and %). The Tanita MC-780 (Tanita Corporation. Tokyo, Japan) estimates muscle mass based on whole body conductivity and uses a conversion equation that is calibrated with dual-energy X-ray absorptiometry as a reference [23]. Height was measured using a standardised protocol consistent with commonly used anthropometric guidelines [24]. We used a tape measure while participants stood barefoot against a wall, ensuring contact with the heels, back, and head.

#### 2.3.2. Physical Fitness

A trained researcher assessed physical fitness using the ID-fitscan, a standardised set of performance tests for adults with ID [25]. It includes tests for lower body functional muscular endurance (30 s chair stand and 5-times chair stand) [26], general muscle strength (grip strength by hand-held dynamometry) [27], and balance (four static stances, comfortable gait speed).

For the 30 s chair stand, participants stood up and sat down as often as possible in 30 s, without using their hands. We recorded the number of complete stands. For the 5-times chair stand, participants stood up and sat down five times as fast as possible, without using their hands. We recorded the time needed to complete five stands.

Grip strength was measured in a seated position (shoulder 0° flexion, elbow 90° flexion) using the Jamar dynamometer (Hydraulic, JAMAR, Sammons Preston Rolyan, Nottinghamshire, UK). The participants were asked to squeeze with maximum force three times with both hands, with one minute rest in between. The highest value of six attempts (kg) was reported.

Static balance was measured with four stances, progressing in difficulty (side-by-side stand, semi-tandem stand, tandem stand, and single leg stand), which the participants had to maintain independently for 10 s. A maximum of five attempts was allowed. We recorded the time for each completed stance and used the scores to categorise static balance from 0 (worse) to 4 (best) [28].

Dynamic balance was measured as comfortable gait speed over the middle five metres of an 11 m walkway. After three-metres for acceleration, the time between the three metre and eight-metre mark was measured using a stopwatch. The last three metres were used for deceleration. We converted the average time of the three trials (s) to gait speed (m/s).

#### 2.3.3. Sarcopenia

We used the European cut-off values (Table 1) to classify participants as non-sarcopenic, sarcopenic, or severe sarcopenic [8]. They were classified with sarcopenia when they scored below the cut-off value for grip strength or above cut-off value for the 5-times chair stand and below cut-off value for muscle mass. Participants were classified with severe sarcopenia when they also scored below the cut-off for physical performance, with a comfortable gait speed ≤ 0.8 m/s.

#### 2.3.4. Activities of Daily Living

Activities of daily living were assessed with the Barthel Index for basic activities of daily living [29] and an adapted version of the Lawton scale for instrumental activities of daily living (IADL) [30]. We incorporated items from the Groningen Activity Restriction Scale (GARS) [31], which was adapted to be used in adults with ID [32]. Items were scored on a 4-point rating scale from 0 (complete help) to 3 (independent) and summed to get a total score.

The Barthel Index includes ten items (bowel/bladder control, grooming, toilet use, feeding, transfer, walking, dressing, climbing stairs, and bathing), with item scoring categories ranging from two to four categories. The total score ranged from 0 (completely dependent) to 20 (completely independent) [33].

#### 2.3.5. Challenging Behaviour

Challenging behaviour was assessed with the Aberrant Behaviour Checklist (ABC) [34], a 58-item questionnaire with five subscales: irritability, lethargy, stereotypic behaviour, hyperactivity, and inappropriate speech. Professional caregivers scored the items from 0 (not a problem) to 3 (severe problem), and subscale scores were summed [34]. Each subscale has its own score range: irritability (0–45), lethargy (0–48), stereotypic behaviour (0–21), hyperactivity (0–48), and inappropriate speech (0–12) [35]. Higher scores indicate more severe behavioural problems.

#### 2.3.6. Falls

A fall was defined as an unintentional event resulting in a person coming to rest on the floor, or other lower level [36]. Participants or their professional caregivers recorded falls daily on a calendar during the 12-week baseline and 12-week follow-up period. Multiple falls in a day were recorded as separate events. For each period, we classified participants as non-fallers or fallers (≥1 fall), and the total number of falls was recorded. This categorisation was chosen to facilitate interpretation of fall occurrence and was used descriptively in the present study.

### 2.4. Intervention

The duration of the PRET intervention [7] was 24 weeks and consisted of two sessions per week, with at least 48 h between the sessions. The intervention was led by experienced physiotherapists and physical activity instructors. The instructor–participant ratio was 1 to 1 or 1 to 2. Prior to the study, all instructors were trained in the intervention protocol, exercises, and standardised reporting procedures. Attendance, progression, and adverse events were recorded in training logbooks.

The intervention consisted of four mesocycles of six weeks (Table 2), following ACSM guidelines [37]. Training intensity was prescribed as a percentage of the estimated one-repetition maximum (1RM), which was calculated from a 10-repetition maximum (10RM) test performed at the start of each mesocycle to ensure progressive overload according to the RESID protocol [18,19]. Each session consisted of seven exercises (RESID) targeting all large muscle groups (i.e., lower body, upper body, and trunk muscles), performed using functional exercise equipment [18]. For each muscle group, alternative exercises were provided for participants who could not perform the original exercise with a correct execution. A detailed description of the intervention protocol, including the included exercises, equipment, and progression scheme, has been published elsewhere [7].

To maximise attendance and motivation, each participant received a progression logbook to track their progress and a T-shirt to wear during the training sessions and measurements. Upon completion of the intervention, participants received a certificate and a medal.

### 2.5. Follow-Up Period

The follow-up period lasted 12 weeks. Immediately post-intervention, all participants were invited to complete a questionnaire assessing their experience with the PRET intervention programme. Although the structured training as part of the study ended, interviews were conducted with participants and trainers who expressed an interest in continuing training. The aim of this interview was to support the integration of physical activity into daily life, outside the research setting.

### 2.6. Statistical Analysis

We used descriptive statistics to present participant characteristics at T0 and post-intervention at T3 and for training logbook data. The same statistical analyses as those used for the primary outcomes of the PRET study were used [19]. We used multilevel linear regression models to investigate changes in all outcomes from baseline to the end of the intervention period (T3). The participant identification numbers were used as a level in the models. This level consisted of the repeated measurements nested within a participant. The three baseline measurements (T0–T2) were included as repeated observations within the participant-level models and jointly represented the baseline period against which post-intervention (T3) and follow-up (T4) outcomes were compared. Due to a large number of trainers, we could not include the level of clustering of participants by trainer in the final model. We explored confounding by change in physical activity level, and effect modification for level of ID, age, and sex. Physical activity levels were assessed throughout the study using a questionnaire and were initially considered as a potential confounder. However, due to substantial missing data and large variability in the responses, these data were considered insufficiently reliable and were therefore not included in the final analyses.

Using the same analytical approach as described above, we separately analysed the changes from baseline (T0, T1, T2) to post-intervention (T3) first, and second, the changes from post-intervention (T3) to 12-week follow-up (T4). We conducted intention-to-treat (ITT) analyses with the data from all participants who started the study. To account for participant dropout and missing follow-up measurements, last observation carried forward was used for missing data in the ITT analyses and to preserve the full study sample and maintain consistency with the predefined statistical analysis plan of the PRET study [7]. For each outcome measure, participants were included in the analysis if the corresponding baseline data were available. Subsequently, we conducted per-protocol (PP) analyses with the data from the participants who completed the intervention. Regression coefficients (β) and their corresponding 95% confidence intervals were reported as the primary measures of the magnitude of observed changes.

To evaluate changes in fall incidents between the baseline (T2) and follow-up (T4), two non-parametric tests were applied, as the data were paired and not normally distributed. We used the Wilcoxon signed-rank test to compare the number of falls per participant across the two periods. Changes in fall status (faller/non-faller) between baseline and follow-up were assessed using the McNemar’s test for paired nominal data.

Statistical significance was set at *p* < 0.05 for all analyses. Statistical analyses were performed with R-statistics (R Foundation for Statistical Computing, Vienna, Austria) and the package Linear and Nonlinear Mixed Effects Models (nlme) [38].

## 3. Results

In total, 36 adults with ID and CVRFs participated in the study. The mean age was 55.6 years (SD = 12.9 years) and 52.8% were male (Table 3). Throughout the study, 10 participants dropped out, resulting in a PP sample of 26 participants who completed the intervention. Reasons for dropout included re-evaluation of decision to participate (n = 1), motivational reasons (n = 7), and medical reasons not related to the intervention (n = 2). There were no significant differences in characteristics (age, sex, level and aetiology of ID) between dropouts and participants who completed the intervention (all *p* > 0.05).

Two participants did not have any completed questionnaires for ADL and challenging behaviour resulting in an ITT sample of 34 for these questionnaires. Within the PP sample, only 19 participants had completed questionnaires.

None of the participants in the PRET study were classified with sarcopenia or severe sarcopenia. Therefore, no analyses could be conducted for sarcopenia. Mean baseline and post-intervention scores for each outcome are presented in Table 4. Results of the multilevel linear regression models for post-intervention and follow-up changes are presented for each outcome in Table 5.

### 3.1. Changes Following the Intervention

#### 3.1.1. PRET Intervention

Trainer-completed logbooks indicated that training load was progressively increased, either by introducing more challenging exercises or by increasing the lifted weight. Participants missed an average of 4.6 out of 48 scheduled training sessions (SD = 3.6), corresponding to an average attendance rate of 90.4%. Within the ITT sample, 37.1% achieved the maximum training intensity of 80% 1RM, and 31.4% reached 75% 1RM. In the PP sample, 52% achieved 80% 1RM and 44% reached 75% 1RM, while 4% did not exceed 65% 1RM.

#### 3.1.2. Body Composition

One participant could not complete BIA measurements due to ataxia and was unable to stand still throughout the measurement. Only weight and height were measured for this participant and BMI was calculated manually. Two participants did not have post-intervention BIA measurements. This resulted in an ITT sample of 35 participants for fat mass and SMM, and a PP sample of 24 participants for weight and BMI and 23 participants for fat mass and SMM. A graphical representation of the mean body composition outcomes across all assessment time points (T0–T4) in the ITT sample is provided in Figure S1. Both the ITT and PP analyses showed no statistically significant changes in any of the BIA measurements following the intervention.

#### 3.1.3. Physical Fitness

In the ITT sample, one participant could not follow instructions to complete measurements for handgrip strength. Within the PP sample, two participants did not complete the post-intervention physical fitness test measurements. Of these two participants, one did not want to perform the tests, and the other was not physically able to perform the tests, resulting in a PP sample of 24.

Mean physical fitness outcomes across all assessment time points (T0–T4) in the ITT sample are presented in Figure S2. Our results from the ITT sample showed a statistically significant improvement in the five-times chair stand with a reduction of −2.40 s (95% CI [−4.06; −0.75]). No other significant changes were found in our ITT sample for physical fitness. In the PP sample, we found statistically significant positive changes in the 30 s chair stand with an increase in repetitions of 0.95 (95% CI [0.05; 1.88]), and in the five-times chair stand with a reduction of −2.54 s (95% CI [−4.86; −0.22]). We found no statistically significant changes in grip strength, gait speed, or static balance in our PP sample.

#### 3.1.4. Activities of Daily Living

A graphical representation of the mean ADL outcomes across all assessment time points (T0–T4) in the ITT sample is provided in Figure S3. We found no statistically significant changes on the outcomes of IADL or BADL in both the ITT and PP samples following the PRET intervention.

#### 3.1.5. Challenging Behaviour

Changes in mean ABC outcomes over time (T0–T4) in the ITT sample are illustrated in Figure S4.

For challenging behaviour, our ITT results showed statistically significant positive changes in the intervention, reflected by decreased scores on irritability with −1.10 (95% CI [−2.21; −0.000]) and inappropriate speech with −0.75 (95% CI [−1.22; −0.28]). We found no statistically significant changes in lethargy, hyperactivity, or stereotypy. Our PP results showed statistically significant improvements, reflected in decreased scores in irritability of −2.13, (95% CI [−3.67; −0.60]), lethargy of −2.37 (95% CI [−3.83; −0.90]), hyperactivity of −1.89 (95% CI [−3.64; −0.14]), and inappropriate speech of −0.94 95% CI [−1.66; −0.21]).

#### 3.1.6. Follow-Up

During the follow-up period, our ITT analyses showed a statistically significant increase on the 30 s chair stand of 0.44 s (95% CI [0.02; 0.87]) from post-intervention to 12 weeks post-intervention. Our PP analyses showed a statistically significant decline on IADL with −1.56 (95% CI [−2.68; −0.43]). We found no further changes in any of the other outcomes during the follow-up period for both ITT and PP analyses.

#### 3.1.7. Falls

Fall agendas were completed by 20 participants. At baseline, eight participants were fallers (five with one fall, one with two, one with six, and one with seven falls). At follow-up, six participants were fallers (three with one fall, two with two, and one with six falls). No statistically significant differences were observed in the proportion of fallers (McNemar *p* = 0.69) or the number of falls (Wilcoxon *p* = 0.47) between baseline and the follow-up period.

**Table 5:** Multi-level regression analyses.

Intention-to-Treat		Post-Intervention				Follow-up			
Outcome	N	β	Intervention	*p*	95% CI	β	Follow-up	*p*	95% CI
BIA									
Weight (kg)	36	86.02	−0.80	0.13	−1.82; 0.22	85.22	−0.43	0.17	−1.05; 0.19
BMI	36	30.13	−0.33	0.12	−075; 0.09	29.80	−0.15	0.16	−0.37; 0.06
Fat mass (kg)	35	28.36	−1.03	0.19	−2.56; 0.50	27.33	−0.36	0.44	−1.28; 0.57
Fat mass (%)	35	31.55	−0.48	0.22	−1.25; 0.29	31.07	−0.72	0.06	−1.45; 0.01
SMM (kg)	35	29.99	−0.11	0.78	−0.90; 0.68	29.88	0.94	0.18	−0.45; 2.33
SMM (%)	35	36.01	−0.47	0.49	−1.81; 0.86	35.54	1.46	0.18	−0.67; 3.59
Physical fitness									
Chair30 (repetitions)	36	12.87	−0.56	0.74	−3.91; 2.79	12.68	0.44 *	0.05	0.02; 0.87
Chair5 (sec)	36	15.79	−2.40 *	0.005	−4.06; −0.75	13.39	0.20	0.76	−1.10; 1.50
Hand grip strength (kg)	35	30.40	−0.43	0.68	−2.48; 1.63	29.97	−0.49	0.51	−1.95; 0.95
Balance	36	3.78	−0.25	0.07	−0.52; 0.02	3.53	−0.14	0.39	−0.46; 0.18
Gait Speed (m/s)	36	1.11	0.04	0.22	−0.02; 0.09	1.15	0.01	0.67	−0.05; 0.07
ADL									
IADL	34	15.19	0.46	0.17	−0.20; 1.12	15.66	−0.63	0.19	−1.57; 0.32
BADL	34	17.19	−0.25	0.07	−0.51; 0.01	16.94	0.38	0.16	−0.15; 0.89
ABC									
Irritability	34	7.63	−1.10 *	0.05	−2.21; −0.00	6.53	0.66	0.32	−0.66; 1.97
Lethargy	34	8.61	−0.76	0.25	−2.06; 0.54	7.84	0.72	0.44	−1.11; 2.54
Stereotypy	34	1.64	0.14	0.67	−0.51; 0.80	1.78	0.19	0.60	−0.53; 0.91
Hyperactivity	34	7.11	−1.15	0.08	−2.43; 0.14	5.97	0.28	0.72	−1.28; 1.84
Inappropriate speech	34	2.99	−0.75 *	0.002	−1.22; −0.28	2.25	0.03	0.87	−0.34; 0.40
**Per Protocol**		**Post-Intervention**				**Follow-Up**			
Outcome	N	β	Intervention	*p*	95% CI	β	Follow-up	*p*	95% CI
BIA									
Weight (kg)	24	83.01	0.19	0.58	−0.49; 0.88	83.21	−0.46	0.32	−1.38; 0.46
BMI	24	28.47	−0.07	0.60	−0.34; 0.20	28.40	−0.06	0.62	−0.30; 0.18
Fat mass (kg)	23	25.65	−0.42	0.50	−1.63; 0.80	25.23	−0.30	0.67	−1.68; 1.09
Fat mass (%)	23	29.50	0.29	0.55	−0.66; 1.24	29.79	−0.91	0.11	−1.99; 0.18
SMM (kg)	23	30.23	−0.09	0.87	−1.07; 0.91	30.25	1.28	0.26	−0.95; 3.52
SMM (%)	23	37.04	−0.71	0.54	−2.97; 1.55	36.38	2.09	0.22	−1.30; 5.49
Physical fitness									
Chair30 (repetitions)	24	11.68	0.95 *	0.04	0.05; 1.86	12.87	0.65	0.06	−0.00; 1.30
Chair5 (sec)	24	14.86	−2.54 *	0.04	−4.86; −0.22	12.35	0.04	0.97	−2.07; 2.15
Hand grip strength (kg)	24	29.52	0.90	0.47	−1.53; 3.33	30.42	−0.71	0.51	−2.86; 1.44
Balance	24	3.95	−0.21	0.22	−0.54; 0.12	3.76	−0.20	0.34	−0.62; 0.22
Gait Speed (m/s)	24	1.18	0.03	0.38	−0.04; 0.11	1.21	0.02	0.64	−0.07; 0.11
ADL									
IADL	19	15.23	0.83	0.17	−0.36; 2.02	16.05	−1.56 *	0.01	−2.68; −0.43
BADL	19	17.65	−0.34	0.14	−0.79; 0.11	17.32	0.12	0.80	−0.78; 1.01
ABC									
Irritability	19	6.87	−2.13 *	0.01	−3.67; −0.60	4.74	1.36	0.20	−0.75; 3.47
Lethargy	19	7.94	−2.37 *	0.002	−3.83; −0.90	5.58	0.89	0.40	−1.22; 2.99
Stereotypy	19	1.88	−0.30	0.52	−1.21; 0.61	1.58	0.02	0.97	−1.18; 1.22
Hyperactivity	19	5.89	−1.89 *	0.04	−3.64; −0.14	4.00	0.80	0.46	−1.36; 2.95
Inappropriate speech	19	2.62	−0.94 *	0.01	−1.66; −0.21	1.69	0.33	0.28	−0.28; 0.94

BIA: bio impedance analysis; BMI: body mass index; SMM: skeletal muscle mass; ADL: activities of daily living; IADL: instrumental activities of daily living; BADL: basic activities of daily living; ABC: aberrant behaviour checklist; N: number of participants in analysis; β: value; CI: confidence interval; sec: seconds; *: statistically significant.

## 4. Discussion

The aim of this study was to investigate changes in body composition, physical fitness, sarcopenia, ADL, challenging behaviour, and falls following participation in the PRET intervention. Significant improvements in the five-times chair stand and challenging behaviour were observed following the PRET intervention. We did not find any statistically significant changes for body composition, the other fitness components, ADL, or falls, and no cases of sarcopenia were observed.

### 4.1. Physical Fitness

Regarding our results for physical fitness, statistically significant improvements were observed in the PP sample in both the 30 s chair stand and five-times chair stand. This indicates that PRET is associated with improvements in lower body functional muscular endurance.

The PRET intervention consists of progressive resistance training, with intensity increasing over time. Logbook records confirmed participants could complete exercises at higher intensities, indicating improved muscular strength. Although lower body functional muscular endurance improved, handgrip strength did not, despite its common use as an indicator of general muscle strength in the general population. This may be explained by the fact that handgrip strength primarily reflects upper limb function. Although the PRET intervention included exercises targeting all muscle groups, it is possible that improvements were more pronounced in the lower extremities, which are not captured by handgrip strength measurements [39]. Although handgrip strength has been found feasible and reliable in this population [40,41], and predictive for a decline in mobility [42] and a higher mortality risk [43], it may not be sensitive for detecting intervention-related changes in general strength. Another explanation is the limited ability of adults with ID to transfer strength gains from exercises to testing tasks, as it requires cognitive and executive functions [15]. This is supported by our findings observed in the chair stand tests, which resembled exercises performed in the PRET intervention, unlike handgrip strength. Additionally, the intervention may have provided an insufficiently specific stimulus for improving grip strength. While PRET targeted all major muscle groups, the exercises were not specifically designed to improve hand or forearm strength.

Furthermore, the ceiling effects might have been an influence for comfortable gait speed and balance, as the included sample was able to walk independently and did not present with physical limitations that could interfere with the intervention. This is reflected in the relatively high baseline values in this study population for comfortable gait speed (1.1 m/s) and static balance (3.8 out of 4.0), suggesting limited room for measurable improvement. For comparison, de Leeuw et al. [4] found an average comfortable gait speed of approximately 0.95 m/s in adults with ID of 50 years and older. These considerations highlight the importance of critically selecting tests that can detect meaningful changes.

### 4.2. Challenging Behaviour

We found significant improvements across multiple domains of challenging behaviour, as presented in both the ITT and PP analyses. This is particularly noteworthy given that participants were not selected based on the presence of behavioural problems, and baseline scores on the ABC subscales were generally low. Even with this limited room for improvement, reductions were still observed. This suggests that resistance training may be beneficial even for individuals with relatively mild behavioural issues. Our findings are in accordance with previous research showing that exercise interventions can reduce the frequency and intensity of challenging behaviour in adults with ID [10]. In contrast to our study, previous studies have typically included samples selected for the presence of challenging behaviour. These findings are consistent with a potential beneficial influence of PRET on challenging behaviour in adults with ID, in addition to its benefits for other health outcomes. The observed improvements may be related to mechanisms previously described in the introduction, including improvements in emotional regulation, executive functioning, and stress resilience following participation in physical activity programmes [14].

Although statistically significant improvements in challenging behaviour were observed, the clinical relevance of these changes remains uncertain, as clinically meaningful change thresholds have not been established for most ABC subscales in adults with ID. Consequently, the statistically significant reductions observed in ABC scores cannot automatically be interpreted as clinically meaningful improvements.

### 4.3. Body Composition

In contrast to previous studies that found modest improvements in adults with ID [20,21], our results showed no statistically significant changes in weight, BMI, fat mass, and SMM. A review by Jacinto et al. reported positive effects of different resistance training interventions on BMI in adults with ID. A more recent study by Gutiérrez-Cruz et al. showed decreases in body mass and body fat mass and an increase in SMM after a 14-week resistance training intervention in adults with ID [21]. However, several differences between our study and those studies may explain the variation in results. While previous studies included adults with ID without specific health issues, the PRET study required the presence of at least two CVRFs for participants to be included. For example, our sample had an average baseline BMI of 30.1 compared to 27.7 in the study by Gutiérrez-Cruz et al. The presence of multiple CVRFs in our sample may have attenuated the potential benefits of PRET. Individuals with multiple CVRFs often present with impaired vascular function [44] and/or metabolic dysregulation [45,46]. These physiological conditions may attenuate or delay adaptive responses to exercise. Furthermore, the study by Gutiérrez-Cruz et al. had a frequency of three sessions per week, compared to two in our study, and statistical analysis was conducted with data from participants who completed at least 12 weeks of the 14-week intervention. Nearly all participants in the study by Gutiérrez-Cruz et al. completed the full 14-week intervention, which indicates better adherence. This higher adherence may have contributed to the more favourable outcomes observed in their study, although this explanation cannot be confirmed based on the present data.

The results found in our study for SMM are consistent with previous research conducted in the general population, showing that initial gains in muscular strength are primarily due to neuromuscular adaptations rather than increases in SMM [47,48,49]. These neural mechanisms have been extensively investigated across various populations. Although such mechanisms have not been specifically studied in adults with ID, previous findings support the notion that an increase in SMM typically occurs after a prolonged period of resistance training [49]. The period of neuromuscular adaptations may be prolonged in adults with ID due to their low levels of physical activity [3], resulting in a greater contribution of these adaptations before increases in SMM occur compared to the general population.

To optimise body composition, diet is an important factor [50]. Reductions in body weight, BMI, and fat mass generally necessitate a calorie deficit [51]. In contrast, stimulating muscle growth, particularly SMM, depends on adequate protein intake [50]. A recent study by Gast et al. [52] revealed that the dietary quality of adults with ID is low. Therefore, as no dietary intervention was included and dietary intake was not assessed in the present study, dietary factors may have influenced the observed changes following participation in the PRET intervention.

### 4.4. Activities of Daily Living

For both BADL and IADL, no statistically significant improvements following the PRET intervention were found. This could be explained by several factors. First, as noted earlier, the transferability of training-related strength gains to daily life tasks may be limited in adults with ID. Second, our sample consisted of participants who were relatively independent at baseline, as indicated by the mean baseline scores for BADL and IADL, which are in the upper range. Therefore, a ceiling effect may have obscured any improvements. This could have contributed to the lack of statistically significant changes observed in ADL, despite evidence from logbooks of increased training load, suggesting an increase in muscle strength.

We found a statistically significant decline in IADL in our PP sample after the follow-up period. This can be explained by an outlier in our data which showed a large unexplained decline of −8 for IADL.

### 4.5. Falls

We found no statistically significant changes in falls. The number of participants classified as fallers decreased slightly from eight at baseline to six at follow-up, and the total number of falls showed a small reduction after the intervention. It is important to note that participants were not selected on fall status. The subgroup of fallers at baseline was small. Consequently, subgroup analyses were not possible, and any true changes within the faller group may have gone undetected.

### 4.6. Sarcopenia

No cases of sarcopenia were identified in the study sample. Since participants were primarily selected based on the presence of CVRFs rather than sarcopenia, this was not unexpected. Additionally, participants were required to have no physical limitations that could interfere with the intervention, which likely reduced the likelihood of sarcopenia within the sample.

However, it is possible that cases of sarcopenia went undetected. While current sarcopenia guidelines [8] rely on fixed cut-off values for muscle mass, these thresholds may underestimate sarcopenia in individuals with obesity [53]. Our sample consisted predominantly of individuals with obesity, which may have impacted the identification of sarcopenia through the cut-off values used for muscle mass in this study. Alternative indicators for muscle mass, such as muscle mass adjusted for weight, have been recommended by The European Society for Clinical Nutrition and Metabolism (ESPEN) and the European Association for the Study of Obesity (EASO) [53]. These indicators may have offered a more accurate representation of sarcopenia. These considerations underscore the importance of critically selecting sarcopenia cut-off values based on the characteristics of the study sample.

### 4.7. Strengths and Limitations

This study provides important insights into the potential clinical applicability of a flexible, pragmatic PRET intervention for adults with ID and CVRFs. The absence of a control group should be considered when interpreting the findings. At the same time, adults with ID represent a highly heterogeneous population with substantial variation in cognitive functioning, physical fitness, health status, and behavioural characteristics. The repeated time series design allowed each participant to serve as their own control, thereby reducing the influence of between-subject variability and enhancing internal validity through within-subject comparisons [54]. Nevertheless, alternative explanations for the observed changes cannot be completely excluded, and causal interpretation should therefore remain cautious.

The intervention was designed to reflect real-world clinical practice, allowing for tailored adaptations based on individual capabilities while maintaining core training principles. These features support the clinical applicability of the intervention in care settings.

We reported effect magnitudes as regression coefficients in the original units of measurement rather than standardised effect sizes. This approach was chosen to facilitate clinical interpretation of the observed changes across outcomes with different measurement scales and to avoid the additional assumptions required when calculating standardised effect sizes in multilevel longitudinal models. Moreover, no single approach to calculating standardised effect sizes in multilevel models has been universally adopted [55]. Because our multilevel longitudinal models included multiple outcomes assessed on different clinical scales, regression coefficients expressed in the original units of measurement were considered more informative than standardised effect sizes. These coefficients provide a direct indication of the estimated change in clinically meaningful units and therefore facilitate the interpretation of the magnitude of change for each outcome. The accompanying 95% confidence intervals provide information on the precision and uncertainty of these estimates.

However, several limitations should be considered when interpreting the results of this study. This report presents secondary outcomes of a study in which the intervention was specifically designed to target the primary outcomes (CVRFs). Therefore, the study population was selected based on the presence of CVRFs, rather than on the presence of behavioural problems, poor fitness, limitations in ADL, fall incidents or conditions such as sarcopenia. Nevertheless, these secondary outcomes remain highly relevant because they are closely related to independence, participation, and quality of life in adults with ID. As participants were not specifically selected based on these domains, the potential for improvement may have been limited, which could have reduced our ability to detect changes associated with the intervention.

The small sample size in this study also limits the ability to detect statistically significant changes. The relatively high dropout rate may have further reduced statistical power despite the use of ITT analyses. In addition, the sample exhibited high heterogeneity in terms of level of ID, physical abilities, and challenging behaviour influencing the outcomes. While including a heterogeneous group enhances the external validity of this study, it complicates the interpretation of the results, as the heterogeneity may have obscured any observed changes, or the observed changes may not apply to all subgroups.

Furthermore, missing data resulting from participant dropout were handled using the last observation carried forward method in the ITT analyses, in line with the predefined analysis plan. Although this method has known limitations and may introduce bias by assuming that participants’ last observed scores remain unchanged after dropout, generally comparable findings were observed in the ITT and PP analyses. Nevertheless, given that 10 of the 36 participants did not complete the intervention, the potential influence of participant dropout and missing data on the findings cannot be completely ruled out.

Several secondary outcomes were evaluated in this study, allowing for a comprehensive assessment of changes observed across multiple domains following participation in the intervention. However, multiple statistical comparisons may have increased the likelihood of type I error. Therefore, these findings should be considered exploratory and should be confirmed in future studies designed to specifically evaluate these outcomes.

### 4.8. Recommendations

Future studies with larger sample sizes are needed to account for the heterogeneity within the population of adults with ID, including differences in cognitive functioning, physical abilities, behavioural characteristics, and support needs. A larger sample size would also increase statistical power and enable to conduct subgroup analyses, which allows for the detection of differential effects according to participant characteristics.

Future research should further explore the effects of resistance training compared to other exercise interventions on challenging behaviour in adults with ID, using samples specifically selected for the presence of challenging behaviour. This approach would allow for a more precise comparison of intervention modalities and help identify which forms of exercise are most effective in reducing challenging behaviour in this population. Challenging behaviour remains one of the most complex and demanding issues in the care for this population, often placing a significant burden on caregivers and support systems [56]. Therefore, identifying effective non-pharmacological interventions is of great importance.

Future studies targeting adults with ID should consider evaluating the feasibility and effectiveness of increased training frequency. While a higher frequency may increase the effectiveness of PRET on muscle strength and body composition [37], it may also lead to an increase in dropout in a population already prone to high dropout rates. Therefore, we recommend conducting comparative studies to assess both the feasibility and the effect of varying training frequencies.

When assessing the effectiveness of PRET in adults with ID, it is essential to critically select outcome measures that are valid, feasible, and sensitive to meaningful changes. Given the heterogeneity within the population, including variations in level of ID and physical functioning, a one-size-fits-all approach is not appropriate. For example, we observed ceiling effects in comfortable gait speed and static balance, limiting the ability to detect improvements over time. This highlights the need for selecting tests that are sufficient for higher-functioning individuals, while still feasible for a variety of levels of ID.

A key strength of the PRET intervention is its adaptability of the exercises to individual capabilities and goals. However, it is crucial to align the exercise selection with the intended outcome it is used for. Therefore, to ensure an effective implementation of PRET and safe execution, it is essential that PRET is delivered by professionals with expertise in both resistance training and working with adults with ID.

## 5. Conclusions

This study examined secondary outcomes of the PRET study in adults with mild or moderate ID and cardiovascular risk factors. Improvements in lower body functional muscular endurance and challenging behaviour were observed following participation in the PRET intervention, while no changes were observed for body composition, activities of daily living, or falls. The clinical relevance of the observed changes in challenging behaviour remains uncertain, as clinically meaningful change thresholds have not been established for most ABC subscales in adults with ID. Given the exploratory nature of these secondary outcome analyses, the findings should be interpreted with caution and confirmed in larger studies.

## Figures and Tables

**Table 1:** Classification for sarcopenia [8].

Domain	Tests	Values
*Sarcopenia*		
Muscle strength	Five-times chair stand	>15 s for 5 rises
	Grip strength	<27 kg (men) <16 kg (women)
Muscle mass	BIA	<20 kg (men) <15 kg (women)
*Severe sarcopenia*		
Muscle strength	Five-times chair stand	>15 s for 5 rises
	Grip strength	<27 kg (men) <16 kg (women)
Muscle mass	BIA	<20 kg (men) <15 kg (women)
Physical performance	Gait speed	≤0.8 m/s

BIA: bioelectrical impedance analysis; kg: kilogram; m/s: meter per second.

**Table 2:** Training parameters according to the ACSM guidelines [37].

Mesocycle	% of 1RM	Sets	Repetitions	Rest
1	-	1–2	20	30 s
2	65	2	15	1 min
3	75	3	10	1 min
4	80	3	8	2 min

ACSM: American College of Sports Medicine; 1RM: one-repetition maximum.

**Table 3:** Participant baseline characteristics (n = 36).

Characteristic	Baseline
Age mean (SD)	
Years	55.6 (12.9)
Sex N (%)	
Men	19 (52.8)
Women	17 (47.2)
Level of ID N (%)	
Severe	1 (2.7)
Moderate	11 (30.6)
Mild	24 (66.7)
Aetiology of ID N (%)	
Congenital	6 (16.7)
Down syndrome	1 (2.7)
Velocardiofacial syndrome	1 (2.7)
Williams syndrome	1 (2.7)
Other	3 (8.3)
Perinatal	3 (8.3)
Unknown	27 (75.0)
Ethnicity N (%)	
Caucasian	32 (88.9)
Afro/American	1 (2.7)
Other	3 (8.3)

ID: Intellectual disability; SD: standard deviation; N: number; %: percentage.

**Table 4:** Mean outcome values at baseline and post-intervention (n = 36).

Outcome	Baseline (T0)	Post-Intervention (T3)
Body composition mean (SD)		
Weight (kg)	86.1 (21.5)	85.2 (19.0)
BMI	30.1 (8.4)	29.8 (7.4)
Fat mass (kg) (n = 35)	28.9 (15.8)	27.3 (13.9)
Fat mass (%) (n = 35)	32.1 (9.8)	30.0 (8.9)
SMM (kg) (n = 35)	29.9 (6.7)	30.2 (6.0)
SMM (%) (n = 35)	35.5 (7.5)	36.2 (7.3)
Physical fitness mean (SD)		
30 s chair stand (n)	10.6 (5.1)	12.5 (4.6)
5-times chair stand (sec)	16.3 (9.4)	12.9 (7.1)
Handgrip strength (kg)	30.5 (11.9)	27 (11.9)
Static balance	3.8 (1.6)	4.0 (1.8)
Comfortable gait speed (m/s)	1.1 (0.4)	1.1 (0.4)
Sarcopenia N (%)		
Sarcopenic	0 (0)	0 (0)
ADL (n = 34) mean (SD)		
IADL	14.9 (9.0)	16.0 (9.2)
BADL	17.3 (3.6)	18.0 (3.7)
Challenging behaviour ABC (n = 34) mean (SD)		
Irritability	7.9 (7.9)	4.0 (6.0)
Lethargy	8.4 (6.7)	4.5 (8.1)
Stereotypy	1.6 (2.3)	0.0 (3.3)
Hyperactivity	7.4 (6.9)	5.0 (5.9)
Inappropriate speech	2.9 (3.3)	1.0 (2.8)
Falls (n = 20) N (%)		
Fallers	8 (22.2)	6 (16.7)
Non-fallers	28 (77.8)	30 (83.3)

BMI: Body Mass Index; SMM: Skeletal Muscle Mass; ADL: Activities of Daily Living; IADL: Instrumental Activities of Daily Living; BADL: Basic Activities of Daily Living; ABC: Aberrant Behaviour Checklist; SD: standard deviation; kg: kilogram; %: percentage; N: number; sec: seconds; m/s: meter per second.

## Data Availability

The datasets generated and analysed in this study are not publicly available due to privacy and ethical restrictions concerning participants with intellectual disabilities. However, anonymised data may be made available from the corresponding author upon reasonable request.

## Supplementary Materials

The following supporting information can be downloaded at https://www.mdpi.com/article/10.3390/sports14080359/s1, Figure S1: Body composition outcomes; Figure S2: Physical fitness outcomes; Figure S3: Activities of daily living (ADL) outcomes; Figure S4: Challenging behaviour.
