# Supplementary material for: Progressive Resistance Exercise Training and Physical and Behavioural Health Components in Adults with Intellectual Disabilities: A Repeated Time-Series Design
Source: Sports (Basel). 2026 Aug 19;14(8):359. doi: 10.3390/sports14080359 (PMC13517735; doi:10.3390/sports14080359)

Figure S1. Body composition outcomes

Mean outcomes from bio impedance analyses across assessment time points (T0-T4) in the intention to treat sample

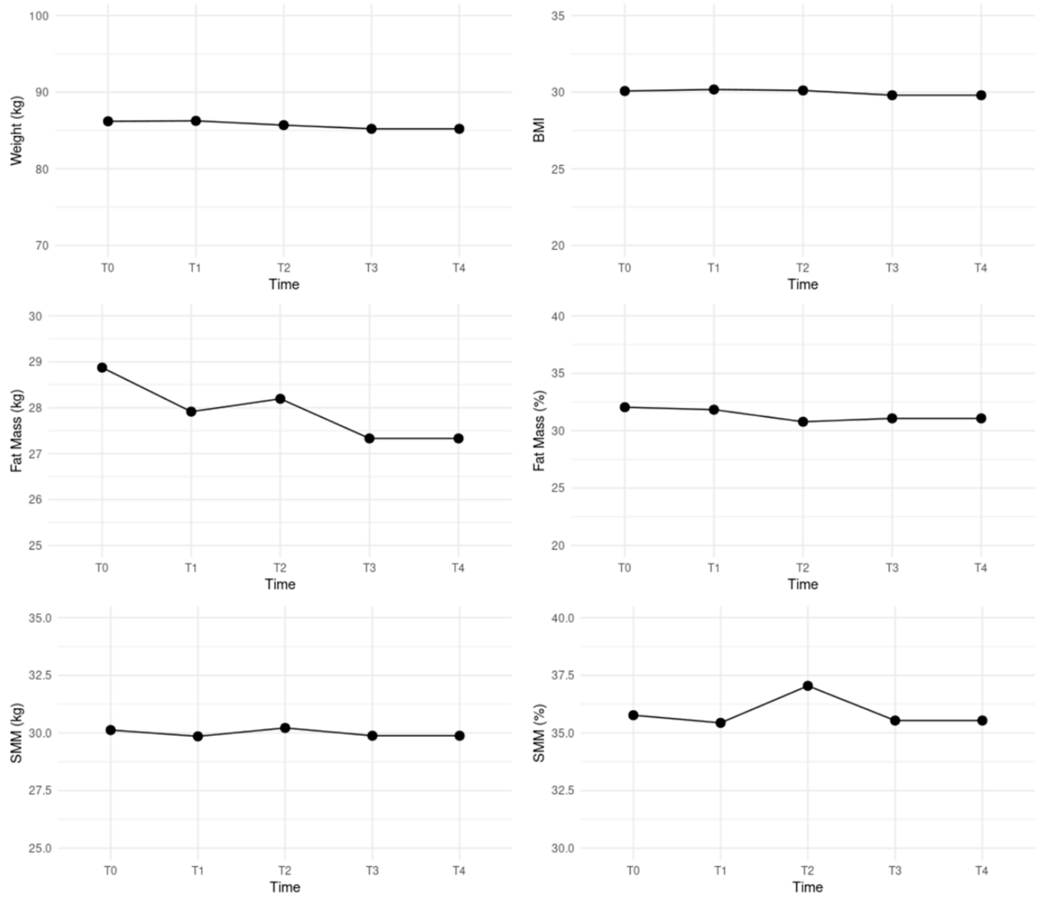

BMI: body mass index, SMM: skeletal muscle mass, kg: kilogram

Figure S2. Physical fitness outcomes

Mean physical fitness outcomes across assessment time points (T0-T4) in the intention to treat sample

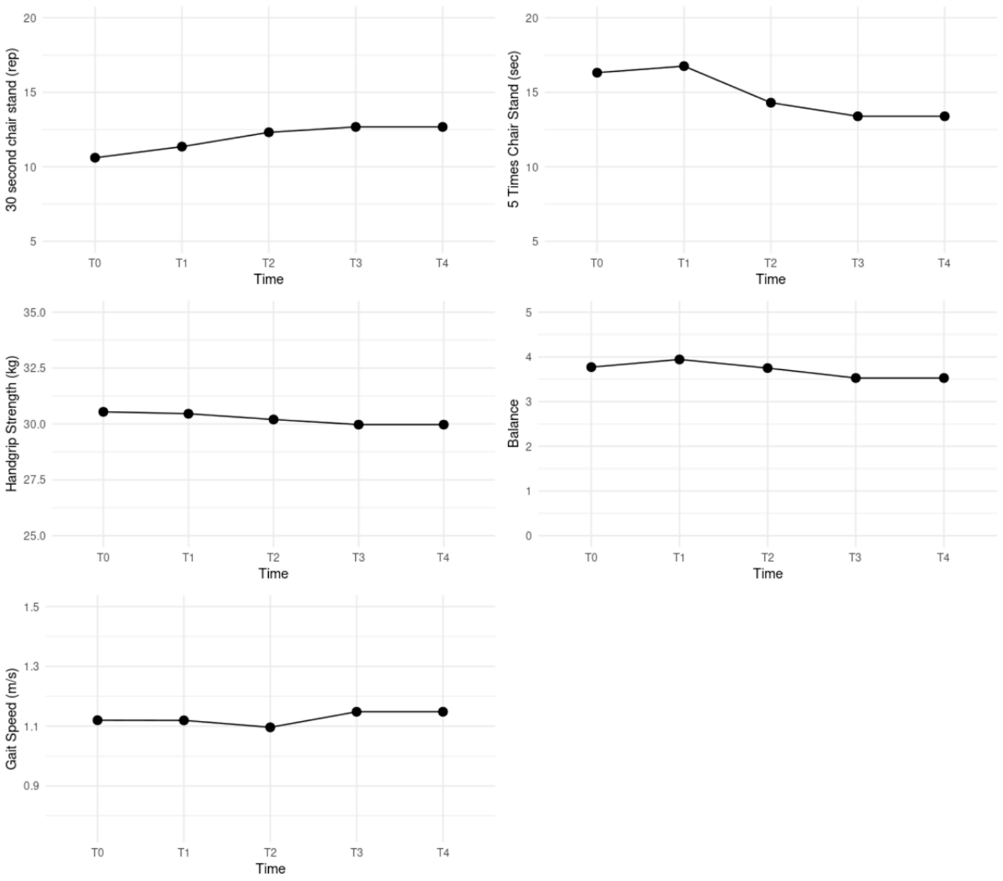

rep: repetitions, sec: seconds, kg: kilogram, m/s: meter per seconds

Figure S3. Activities of daily living (ADL) outcomes

Mean ADL outcomes across assessment time points (T0-T4) in the intention to treat sample

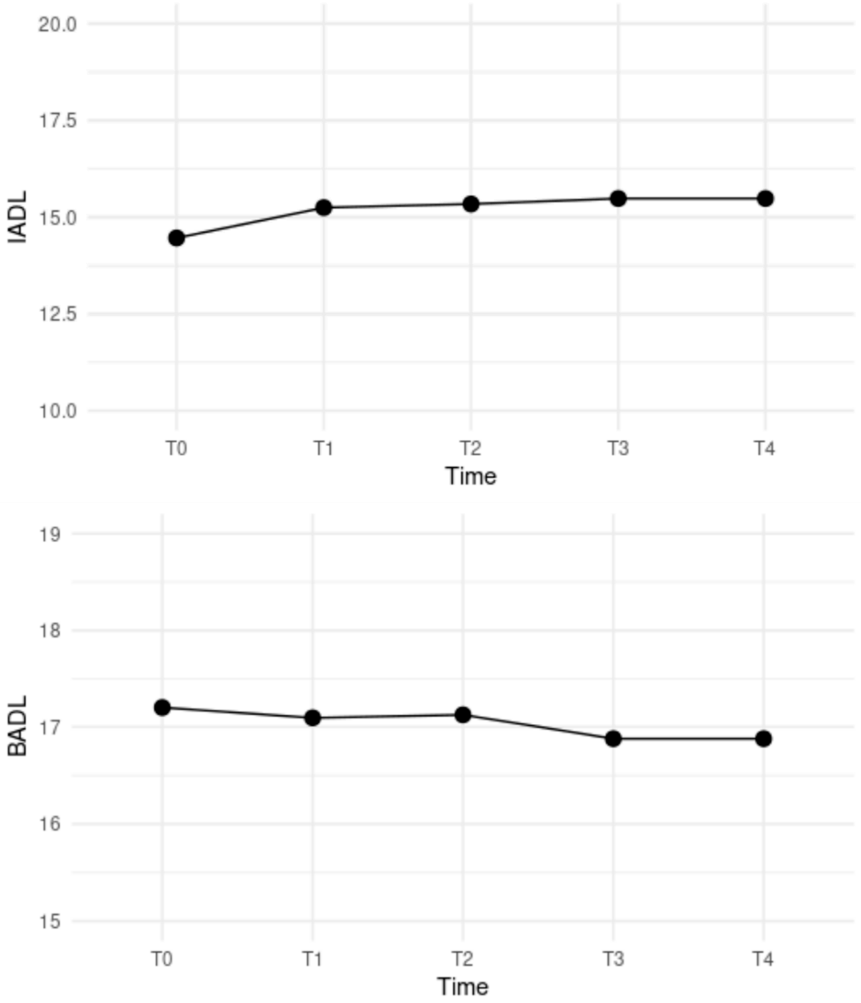

IADL: instrumental activities of daily living, BADL: basic activities of daily living

Figure S4. Challenging behaviour

Mean Aberrant Behaviour Checklist (ABC) outcomes across assessment time points (T0-T4) in the intention to treat sample

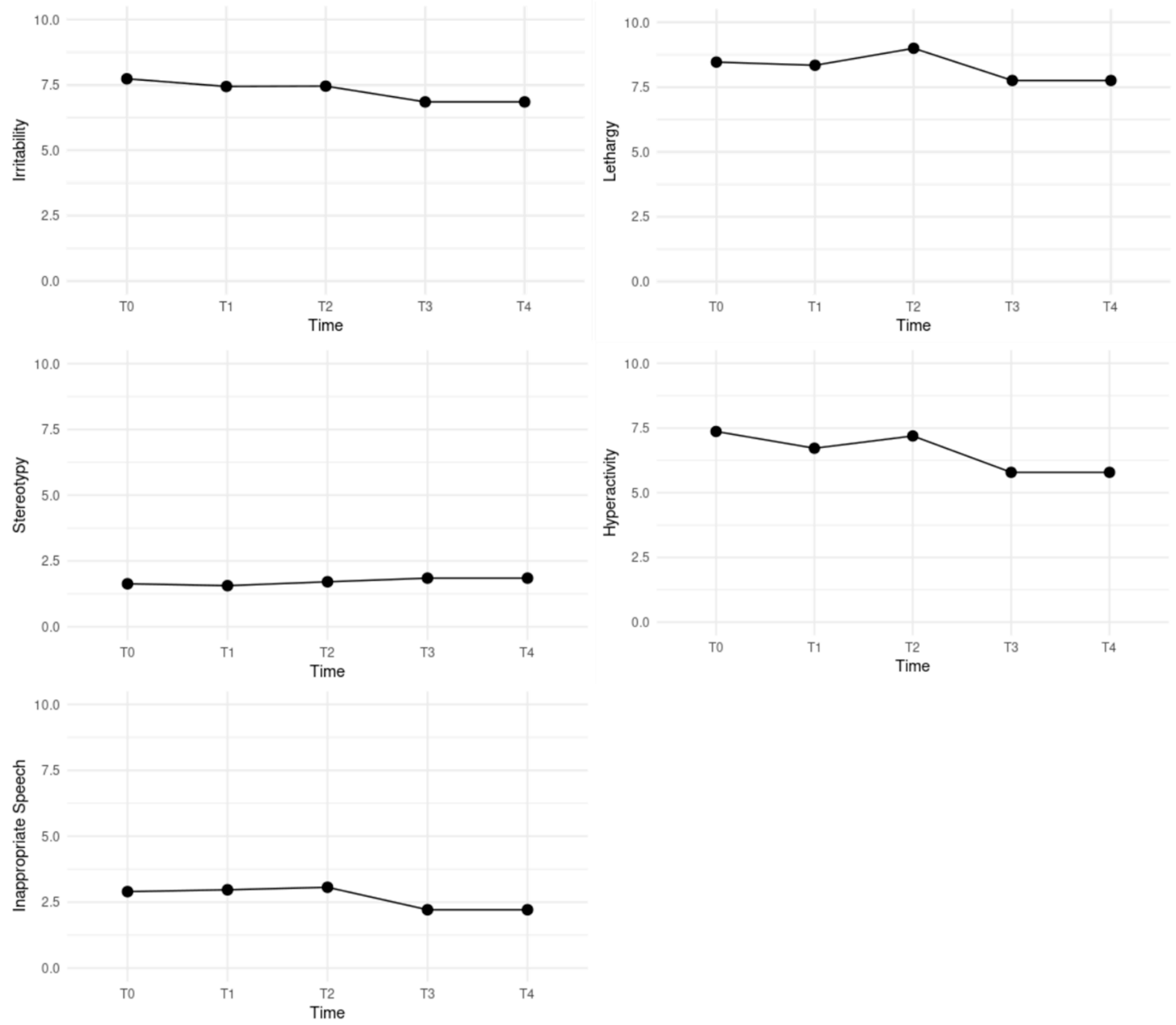

Supplement: Supplementary file 1 [file sports-14-00359-s001.zip › sports-4438676-supplementary.pdf]
